# Fibromyalgia, Pain, and Physical Activity: A Bibliometric Analysis

**DOI:** 10.3390/ijerph20021335

**Published:** 2023-01-11

**Authors:** Ángel Denche-Zamorano, Sabina Barrios-Fernandez, María Mendoza-Muñoz, Jorge Carlos-Vivas, Alejandro Vega-Muñoz, Daniel Collado-Mateo, Pedro R. Olivares, José Carmelo Adsuar

**Affiliations:** 1 Promoting a Healthy Society Research Group (PHeSO), Faculty of Sport Sciences, University of Extremadura, 10003 Caceres, Spain; 2 Occupation, Participation, Sustainability and Quality of Life (Ability Research Group), Nursing and Occupational Therapy College, University of Extremadura, 10003 Cáceres, Spain; 3 Research Group on Physical and Health Literacy and Health-Related Quality of Life (PHYQOL), Faculty of Sport Sciences, University of Extremadura, 10003 Caceres, Spain; 4 Departamento de Desporto e Saúde, Escola de Saúde e Desenvolvimento Humano, Universidade de Évora, 7004-516 Évora, Portugal; 5 Physical Activity for Education, Performance and Health, Faculty of Sport Sciences, University of Extremadura, 10003 Caceres, Spain; 6 Public Policy Observatory, Universidad Autónoma de Chile, Santiago 7500912, Chile; 7 Instituto de Investigación y Postgrado, Facultad de Ciencias de la Salud, Universidad Central de Chile, Santiago 8330507, Chile; 8 Centre for Sport Studies, Rey Juan Carlos University, Fuenlabrada, 28943 Madrid, Spain; 9 Faculty of Education, Psychology and Sport Sciences, Universidad de Huelva, 21007 Huelva, Spain; 10 Facultad de Educación, Universidad Autonoma de Chile, Talca 3480094, Chile

**Keywords:** resistance training, aerobic training, chronic pain, women, cognitive-behavioral therapy

## Abstract

Fibromyalgia (FM) is a rheumatic disease characterized by pain, fatigue, low-quality sleep, depression, anxiety, stiffness, fall risk, mood disturbance, cognitive impairment, poor physical condition, and other symptoms leading to a worse quality of life. Physical activity (PA) and exercise are effective methods to reduce FM symptoms, including pain. This study presents the first bibliometric study on FM, pain, and PA. An advanced search of the Web of Science (WoS) Core Collection database performed on this topic using was carried out traditional bibliometric laws. A total of 737 documents were found. Annual publications presented an exponentially growing trend (R^2^ = 85.3%). Rheumatology International, Kaisa Mannerkorpi, and the USA were the journal, co-author, and country most productive, respectively. The exponential growth of annual publications on FM, PA, and pain shows the high interest of researchers and publishers in this topic. The document “Fibromyalgia A Clinical Review” was the most cited. Moreover, Kaisa Mannerkorpi was the most prolific co-author, Rheumatology International was the most prolific journal, “Fibromyalgia: a clinical review” was the most highly cited document, and Daniel Clauw was the most cited co-author.

## 1. Introduction

Fibromyalgia (FM) is the second most common rheumatic disease after osteoarthritis [1]. Its prevalence differs according to diagnostic criteria [1], although it is estimated that 2–8% of the global population has an FM diagnosis [1,2,3]. In the USA, its prevalence has been estimated at 2–5% [4], while a prevalence of 3.9% is estimated in Europe [2]. FM costs in the US healthcare system reach USD 25 billion annually [5]; in Spain, the annual costs reach EUR 4223 million in the public health system (EUR 11,629 per patient) [2]. FM is more prevalent in women [6,7] and has a severe impact on individuals’ functionality, requiring interventions based on comprehensive and continuous care provided by social, health, and occupational stakeholders [8].

FM is a chronic syndrome characterized by widespread pain and other symptoms such as fatigue, sleeping issues, imbalance, stiffness, higher fall risk, poorer physical fitness, mood disturbance, anxiety, depression, and cognitive impairment [9,10,11], which impacts the individual’s quality of life [12]. FM a etiology and pathophysiology are complex [13]. It has been suggested that abnormalities in central monoaminergic transmission may play an important role, with serotonin- and noradrenaline-mediated descending pain inhibitory pathways being involved in the onset of this pain [14] and with ascending pain pathways upregulated and descending pain inhibitory pathways downregulated [15]. This pathology involves biological, psychological, and social factors [13], and then healthcare professionals may experience difficulties in diagnosing this disease, which can be extended up to 2 years, requiring numerous medical visits, specialists, and trials up to 10 years before diagnosis [11]. Genetic and family predisposition are major risk factors: an eight-fold increased risk has been documented in immediate family members of people with FM compared to those without this diagnosis [15,16]. Poor sleep quality has been documented to lead to FM development as sleep disturbances can impair descending pain inhibitory pathways [17]. Other modifiable risks are overweight and obesity, which are also associated with a worse clinical presentation [18], and stress and other psychological factors, which can induce neural responses that amplify pain perception [15].

Multidisciplinary care based on pharmacological and non-pharmacological treatments is recommended for FM management and its symptoms [19]. On the one hand, pharmacological treatments include tricyclic antidepressants, anti-epileptic drugs, selective serotonin reuptake inhibitors, norepinephrine/serotonin reuptake inhibitors (SNRIs), antidepressants [15,19], antioxidants, vitamins, and natural analgesics, among others [20]. On the other hand, non-pharmacological interventions include psychological treatments, cognitive-behavioral therapy, hydrotherapy, physiotherapy, transcutaneous electrical nerve stimulation (TENS), transcranial direct current stimulation (tDCS), education programs [15,19,21] and physical activity (PA) [13,22,23]. Among the latter, PA has shown positive effects in improving multiple patient-centered outcomes [21], as exercise reduces the symptoms of most musculoskeletal pathologies [22] and people with FM have shown worse physical conditioning [13]. Aerobic exercise, such as fast walking or music-supported aerobic exercise, strength training, or combinations of both, is also effective [24,25,26,27,28]. Traditional exercise programs often have low adherence levels [29] due to psychosocial barriers such as pain, fatigue, bad weather, demotivation, feeling stressed, sad, and worried, and having a bad day because of FM. Therefore, other physical exercise interventions such as the square stepping exercise [30], virtual reality, and exergames [31,32], or exercise in nature [33] could be good alternatives. Health education programs, together with non-pharmacological treatments based on PA, are effective in improving and controlling FM symptoms [30,34] and quality of life [35].

In the scientific bibliography, FM and PA research can be found, including cross-sectional studies [36,37], randomized controlled trials [38,39], systematic reviews and meta-analyses [11,40,41], and scoping reviews [23,42]. However, although other bibliometric analyses on FM exist [43,44,45,46,47], no bibliometric studies related to FM, pain, and PA have been found. The mapping of an area of scientific knowledge through a bibliometric analysis is useful to provide essential information to researchers, such as identifying research opportunities, trends in scientific production, and substantiating scientific research, among other utilities [48]. Thus, this study aims to analyze the trends followed by annual publications on this topic, identifying co-authors and most productive and cited journals, the countries with the highest number of publications, the most cited documents, and the most used keywords.

## 2. Materials and Methods

### 2.1. Design and Data Source

A bibliometric analysis was performed based on the traditional laws of bibliometrics [49] on documents published in journals indexed in the Web of Science (WoS) Database Core Collection of Clarivate Analytic, in their Science Citation Index Expanded (SCI-Expanded), Social Sciences Citation Index (SSCI), and Emerging Sources Citation Index (ESCI) editions. The WoS was chosen as the primary data source as it is one of the most prestigious databases in the scientific community and one of the most widely used for this type of review [45,47,50], providing a large amount of information related to publications: co-authors, title, abstract, keywords, source of publication, publisher, affiliations of co-authors, country/regions, references cited, types of documents, and funding information, among others.

The search was conducted on 6 October 2022, performing an advanced search using the following search vector: ti = (fibromyalgia) and (ti = (“physical activity”) or ab = (“physical activity”) or ak = (“physical activity”) or ti = (exercise) or ab = (exercise) or ak = (exercise) or ti = (“physical training”) or ab = (“physical training”) or ak = (“physical training”) or ti = (“resistance training”) or ab = (“resistance training”) or ak = (“resistance training”)) and (ti = (pain) or ab = (pain) or ak = (pain)). The tags used were ti (searches on article titles), ab (searches on article abstracts), and ak (searches on author keywords). Data were extracted in .xslx format to be analyzed in Microsoft^®^ Excel^®^ for Microsoft 365 MSO version 2206 and in plain text to be analyzed with VoSViewer^®^ 1.6.18.

### 2.2. Statistical Analysis

The fit of the trend followed by the annual publications to an exponential growth ratio was tested (adjusted R^2^) by applying De Solla Price’s law of exponential growth of science [51,52]. A descriptive analysis of the WoS subject categories in which the documents were found was conducted using the WoS Analyze Reports to identify the categories with the highest number of documents, the most cited articles in these categories, and the most productive journals and co-authors. The most productive and most cited journals were highlighted by applying Bradford’s law of concentration [49,53,54]; the most productive co-authors were highlighted using Lotka’s law, applying the Hirsch index (h-index) [55] on the most prolific co-authors to highlight the most prominent (the most cited among the most productive) [49,56]. A descriptive analysis of the most productive countries/regions was performed. The most cited papers were identified using the h-index highlighting outliers in the number of citations [49,55]. Finally, Zipf’s law was applied to find the most used author keywords [57]. Fractionalization and association strength analyses were used to create the visualizations through the VOSviewer software.

## 3. Results

### 3.1. Annual Publication Trends

**Figure 1:**
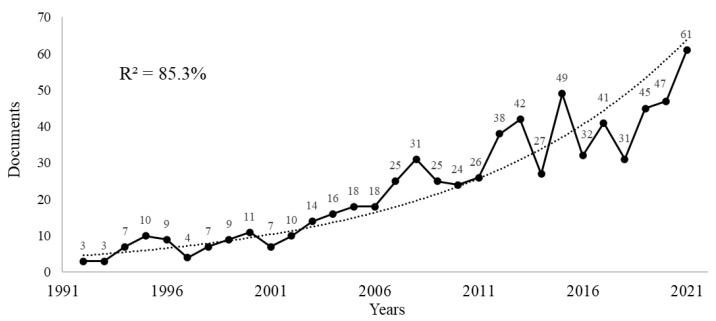
Annual publication trends graph.

A total of 737 documents were found (602 articles and 135 reviews). The first documents, “Reactive fibromyalgia syndrome” [58] and “Living with fibromyalgia: consequences for everyday life” [59], were published in June 1992. From 1992 to 2022 there was a continuity in the annual publications with an exponential growth trend between 1992 and 2021 with R^2^ adjusted at 85.3% to an exponential growth rate (Figure 1).

### 3.2. Web of Science Categories

Papers were assigned to 62 different WoS subject categories, with the most prominent being rheumatology (262 documents), rehabilitation (111 documents), clinical neurology (94 documents), general internal medicine (72 documents), and sport sciences (68 documents). In the rheumatology category, the most cited articles were: “EULAR revised recommendations for the management of fibromyalgia” [11] and “EULAR evidence-based recommendations for the management of fibromyalgia syndrome” [60]. The most productive co-author was Kaisa Mannerkorpi, and Rheumatology International was the journal with the highest number of publications. In the rehabilitation category, the two most cited articles were “Effects of aerobic exercise on pain perception, affect, and level of disability in individuals with fibromyalgia” [61] and “From acute musculoskeletal pain to chronic widespread pain and fibromyalgia: application of pain neurophysiology in manual therapy practice” [62], while the most productive co-author was, again, Kaisa Mannerkorpi, and the Journal of Musculoskeletal Pain was the most productive journal. In the clinical neurology category “Temporal summation of pain from mechanical stimulation of muscle tissue in normal controls and subjects with fibromyalgia syndrome” [63] and “Guidelines on the management of fibromyalgia syndrome—a systematic review” [40] were the most cited documents, with Wilfried Häusser and Pain being the co-author and journal, respectively, that were most productive. In the general internal medicine category, “Fibromyalgia, a clinical review” [1] and “Management of fibromyalgia syndrome” [64] were the most cited documents, in which Julia Bidonde and Angel Busch were the most prolific co-authors, and Cochrane Database Systematic Review and the Journal of Clinical Medicine were the most prolific journals. Last, in the sport sciences category, “Are women with Fibromyalgia less physically active than healthy women?” [65] and “Aquatic training and detraining on fitness and quality of life in fibromyalgia” [66] were the most cited documents, with Inmaculada Álvarez-Gallardo, Víctor Segura-Jiménez, and Manuel Delgado-Fernández as the most productive co-authors and Archives of Physical Medicine and Rehabilitation as the most productive journal.

### 3.3. Publication Titles

The publications were published in 227 journals. Table 1 shows the 15 journals with the highest number of publications. These journals conform to Bradford’s Core journals based on the number of documents, with 31.5% of the documents (232 papers) compared to 33.6% (53 journals and 248 papers) for Bradford Zone I journals and 34.9% (209 journals and 252 papers) for Bradford Zone II journals. Rheumatology International (30 documents) was the journal with the most published documents.

According to the number of citations, Bradford’s Core journals were composed of six journals whose documents were cited 915 times or more, accumulating 34.3% of the citations in journals included in the WoS Core Collection; Bradford’s Zone I was formed by 16 journals, accumulating 32.9% of the citations; and Zone II contained 255 journals, accumulating the remaining 32.7%. Table 2 shows the journals that composed Bradford’s core and Zone I according to the number of citations. The Journal of Rheumatology was the journal with the highest number of citations (2469 citations).

### 3.4. Most Prolific Co-Author

**Figure 2:**
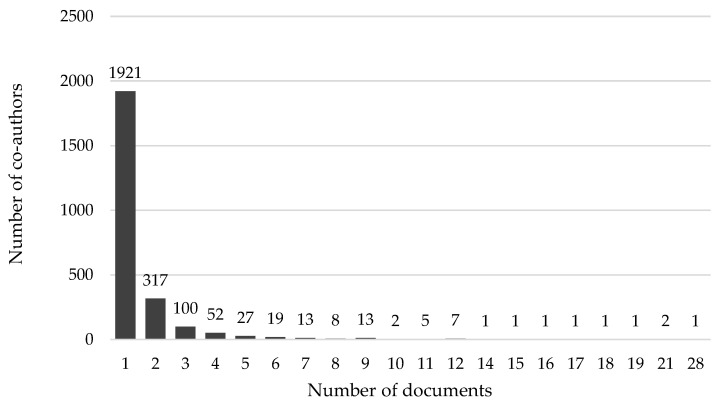
Distribution of co-authors according to the number of publications per co-author.

A total of 2493 co-authors, who had published between 1 and 28 papers, were found. Most of the co-authors had published 1 or 2 papers (2238 co-authors, 90%), with only 1% having published 10 or more (11 co-authors). Figure 2 shows the distribution of co-authors according to the number of published manuscripts.

**Figure 3:**
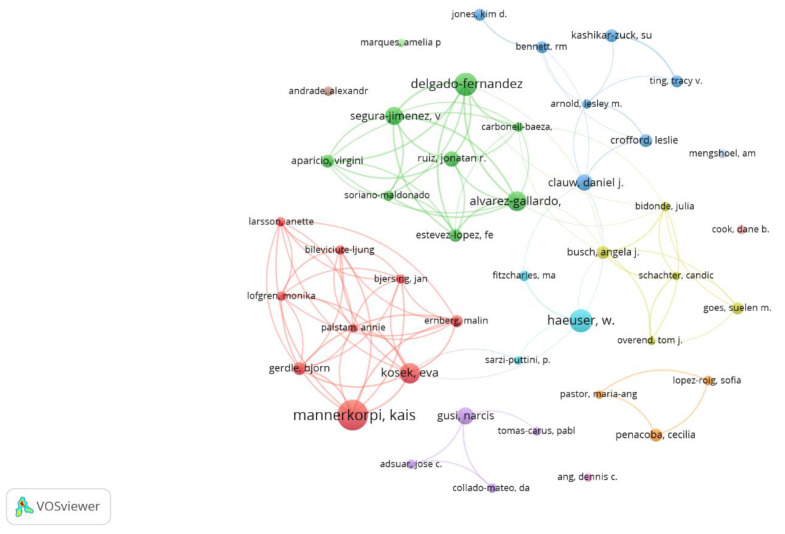
Most prolific co-authors according to the number of documents and publication collaborations.

After applying Lotka’s law, it was estimated that the most prolific co-authors should be, at most, the 49 co-authors with the highest number of papers (square root of 2493). With 57 co-authors with 7 or more papers and 44 co-authors with 8 or more papers, these latter were considered to be the most prolific co-authors. As the 44 most prolific co-authors had 58 or more citations by applying the h-index, they were also considered the most prominent. Kaisa Mannerkorpi (University of Gothenburg, Sweden) was the most prolific co-author, with 28 documents; followed by Winfried Häuser (Technical University of Munich, Germany) and Manuel Delgado-Fernández (University of Granada, Spain), each with 21 documents; Eva Kosek (Karolinska Institutet, Sweden), with 19; and Inmaculada Álvarez-Gallardo (University of Cadiz, Spain), with 18 publications. Figure 3 shows the 44 most prolific co-authors according to the number of publications and their relationships according to their publications (analysis: association strength; attraction: 8; repulsion: −2; node size: documents; color: cluster).

**Figure 4:**
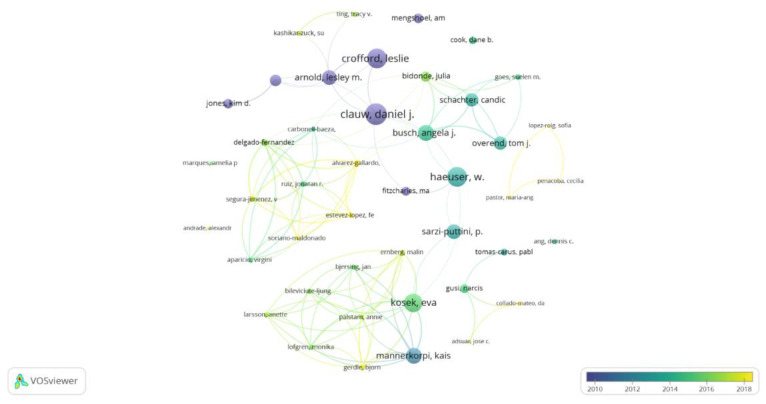
Most prolific co-authors.

Among the 44 most prolific co-authors, Daniel Clauw (University of Michigan, USA) was the most cited co-author, accumulating 2147 citations; the second author was Leslie Crofford (Vanderbilt University, USA), with 1871 citations; the third was Wilfred Häeuser, with 1852 citations; then Eva Kosek, with 1638 citations; and last, Ernest Choy (Cardiff University, Walles), with 1369 citations. Figure 4 shows the 44 most prolific co-authors according to their number of citations and average year of publication (analysis: association strength; attraction: 8; repulsion: −2; node size: citations; color: average publication year).

### 3.5. Countries/Regions

The USA was the country with the highest number of documents (211) and citations (10,132). Spain was the second most productive (143 documents) but the fourth most cited (2617 citations). According to the number of citations, the second most productive country was Sweden (3070 citations, in 46 documents). Brazil (85 documents and 1326 citations), Turkey (49 documents and 1826 citations), and Germany (48 documents and 2432 citations) completed the top 5 most productive countries. In terms of the number of citations, Canada (2923 citations and 46 documents), in third place, and England (2556 citations and 26 documents) completed the top 5 countries.

**Figure 5:**
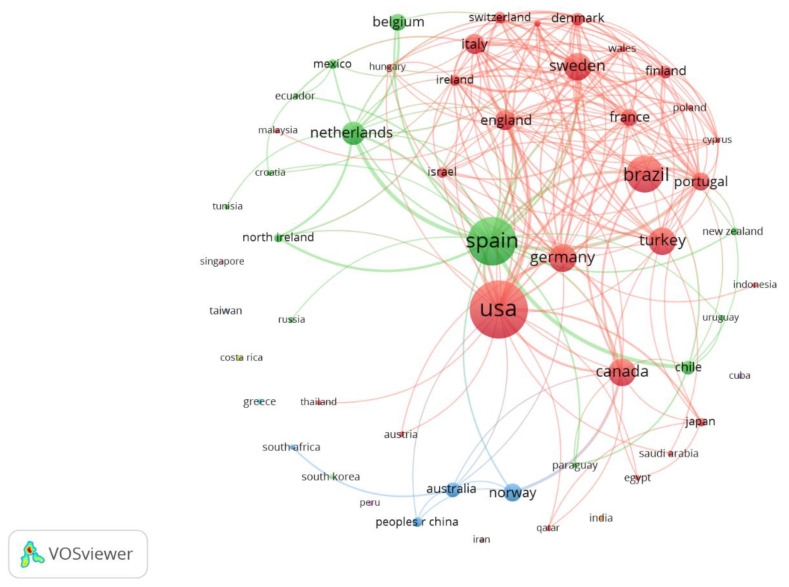
Clusters formed by countries/regions.

Figure 5 shows the clusters formed by countries/regions. The USA led the cluster with the largest number of collaborating countries (28). There were two other large collaboration clusters: one of them formed by 13 countries/regions without a leader and another smaller group was led by Spain together with three other countries/regions (analysis: fractionalization; attraction: 10; repulsion: 0; cluster resolution: 0.5).

### 3.6. Most Cited Documents

**Figure 6:**
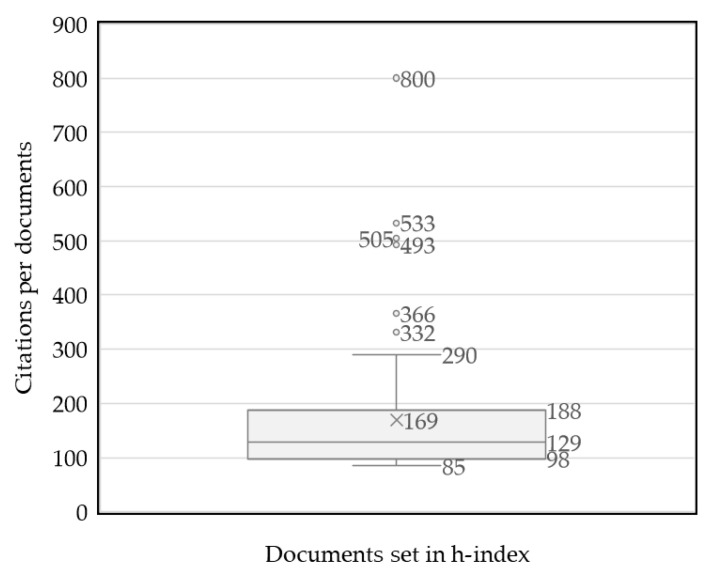
Citations per article on Fibromyalgia and Physical Activity.

After applying the h-index, 81 papers were highlighted as cited 85 or more times, with a citation range from 81 to 800. Among these articles, the citations mean was 169 (median 129). Six papers with a citation count far higher than the rest were the most highly cited: “Fibromyalgia: a clinical review” (800) [1], “EULAR revised recommendations for the management of fibromyalgia” (533) [11], “Management of fibromyalgia syndrome” (505) [64], “EULAR evidence-based recommendations for the management of fibromyalgia syndrome” (493) [60], “Fibromyalgia syndrome: a review of clinical presentation, pathogenesis, outcome measures, and treatment” (366) [67], and “Patient perspectives on the impact of fibromyalgia” (332) [68], with 14 other papers in the upper quartile (Figure 6, Appendix A).

### 3.7. Author Keywords

**Figure 7:**
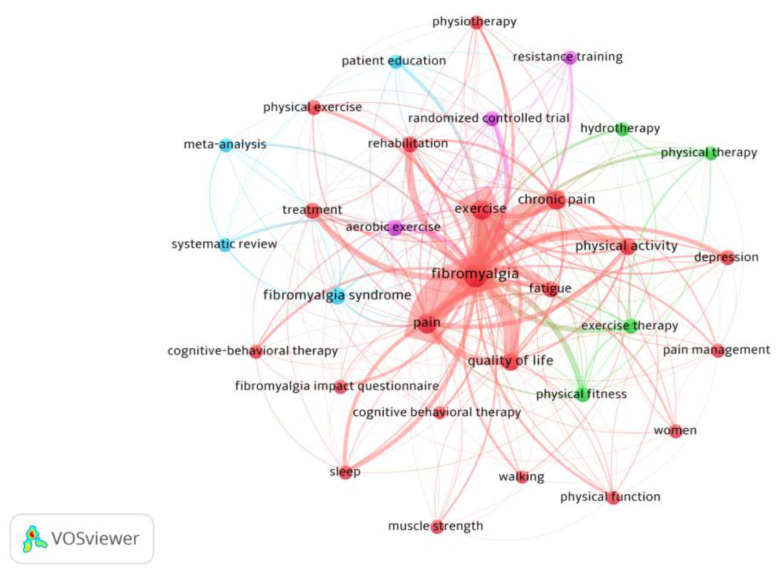
Citations per article on fibromyalgia and physical activity.

After applying Zipf’s law, 32 keywords with 9 or more occurrences were highlighted. Four clusters were identified within the most frequently used keywords (Figure 7; analysis: association strength; attraction: 4; repulsion: −2). The cluster with the most keywords had FM, their symptomatology and affected dimensions (pain, fatigue, chronic pain, depression, quality of life, or sleep) as a central theme as well as central search terms (exercise or physical activity), among others (red cluster). The other clusters had themes related to interventions such as hydrotherapy, physical therapy, exercise therapy (green cluster), research methodologies in fibromyalgia: a meta-analysis and systematic review (blue cluster), and aerobic and resistance training (pink cluster).

**Figure 8:**
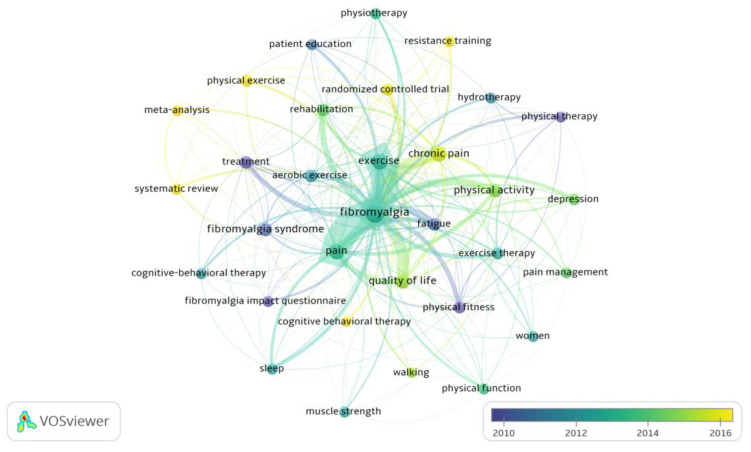
Documents with most frequently used author keywords by average year of publication.

Analyzing the average publication year of the documents in which the most used author keywords appeared, concepts such as strength training, physical exercise, and cognitive therapies were among those of most current interest (analysis: association strength; attraction: 4; repulsion: −2; node size: occurrences; score: average publication years), as Figure 8 displays.

## 4. Discussion

This study presents the first bibliometric analysis based on FM, pain, and PA research. Although other bibliometric studies related to FM exist, they did not comply with traditional bibliometric laws, and they used different methodologies compared with this study. Moreover, the covered topics were different, including FM and biomechanics, FM research, juvenile primary FM syndrome, acupuncture, and the top 100 Cited Articles on Fibromyalgia Syndrome [43,44,45,46,47].

Publications on FM, pain, and PA followed an exponential growth trend from 1992 to 2021, reaching the highest number of annual publications in 2021 (61 documents). At the time of this analysis, 46 papers had already been published in 2022, so this topic arouses high interest among researchers and journals. A similar growth was found in the bibliometric study on acupuncture and FM [46], reaching its highest number of annual publications in 2020 (28 documents). This interest was also reported in the JPFS bibliometrics [45], although the number of annual publications declined from its maximums in 2008 and 2010 (6 documents) to the present, similar to FM and biomechanics bibliometrics [43], with irregular annual publication numbers. The growing interest in research related to FM and PA had already been found in this study by Ortega-Martín et al. [43]. This interest was justified by the evidence found in numerous studies on the effectiveness of muscle strengthening, stretching, and aerobic exercises in improving symptomatology in people with FM, according to other bibliometric studies on FM [43,47,69]. In line with other bibliometric studies, the USA was identified as the most productive country [45,46], showing strong global collaboration networks with other countries such as Spain or Brazil and a large number of productive researchers and research groups in the field. Since FM is a rheumatological disease [1], rheumatology-related journals showed great interest in FM, pain, and PA studies. The three most productive journals on the topic were: Rheumatology International, Clinical and Experimental Rheumatology, and the Journal of Rheumatology, in line with previous bibliometric studies [45,47]. These three journals, along with other rheumatology-related journals, represent great options for researchers to submit their manuscripts on FM, pain, and PA. Journals related to arthritis or pain may also be suitable options as FM is a syndrome difficult to diagnose [1,60,64], with its main symptom being central nervous system pain amplification [1,70], while pain response to touch in muscles and tendons suggests the abnormal sensitivity of nociceptors [63]. Fatigue, memory problems, insomnia, and mood disturbances are the other main symptoms described in the bibliography. Therefore, symptom management is one of the most interesting issues for researchers according to the most cited documents [1,11,60,64]. Additionally, comorbidities, including depression and anxiety [68,71,72], as well as the study of quality of life, are the main issues discussed in the research [39,73,74]. Moreover, PA and exercise (aerobic exercise, strength training, aquatic exercise, and yoga, among others) have proved to be effective in people with FM when measured with tools such as the Fibromyalgia Impact Questionnaire (FIQ) [75,76]. Thus, PA benefits included positive effects on pain [77,78,79,80], depressive symptoms [30], and sleep issues [80,81]. Despite the mentioned benefits, pain, fatigue, psychological discomfort, or lack of time appear as major barriers that limit adherence to exercise programs, representing a major challenge [82,83,84] and being one of the reasons for the researchers to continue their research investigating innovative, more motivating, and less time-consuming PA programs using new technologies and other exercise-based activities for people with FM [32,39,73,85].

This study’s practical implications are related to deepening the knowledge of researchers and publishers interested in the topic, based on the traditional laws of bibliometrics. Annual publications on FM, pain, and PA are in an exponential growth phase, which ensures a large critical mass of researching and publishing on this topic. The most productive journals, authors, and collaborative networks, as well as lines of research and topics of recent interest, have been identified. The information in this study favors the collaboration between researchers, facilitating the location of experts on the topic and relevant documents and journals interested in the manuscripts derived from the different research projects. The most important limitation was the selection bias committed by obtaining data only from the WoS: despite being the most widely used database for this type of study, future lines should include other databases.

## 5. Conclusions

Research on FM, pain, and PA is experiencing exponential growth, so this subject generates great interest. The positive effects found in multiple studies on non-pharmacological treatments based on PA and exercise are one of the reasons for the proliferation of new research related to this topic.

The USA and Spain were the most productive countries and Kaisa Mannerkorpi and Rheumatology International were the most prolific co-author and journals, respectively. “Fibromyalgia: a clinical review” was the most highly cited document, while Daniel Clauw was the most cited co-author.

## Figures and Tables

**Table 1:** Bradford’s Core journals according to the number of documents.

Bradford’s Zone	Journals (Publisher)	N° Doc.	%Doc.	Cit.	JIF	Q.	%O.A.
Core	Rheumatology International (Springer)	30	4.1	915	3.580	Q3	18.1
	Clinical & Experimental Rheumatology (Clinical & Experimental Rheumatology S.A.S.)	24	3.3	905	4.862	Q2	0
	Journal of Rheumatology (The Journal of Rheumatology Publishing Co. Ltd.)	23	3.1	2469	5.346	Q2	1
	Journal of Musculoskeletal Pain (Taylor & Francis)	21	2.8	198	n.a.	n.a.	n.a.
	Arthritis Care & Research (Wiley)	15	2	548	5.178	Q2	13.5
	Clinical Rheumatology (Springer)	15	2	445	3.650	Q3	10.8
	Pain (Lippincott Williams & Wilkins)	15	2	1557	7.926	Q1	16.8
	Archives of Physical Medicine and Rehabilitation (Elsevier)	12	1.6	395	4.060	Q1	6.2
	Clinical Journal of Pain (Lippincott Williams & Wilkins)	12	1.6	741	3.423	Q2	9.3
	International Journal of Environmental Research and Public Health (MDPI)	12	1.6	50	4.614	Q1	95
	Journal of Back and Musculoskeletal Rehabilitation (IOS Press)	12	1.6	66	1.456	Q4	1.1
	Arthritis & Rheumatism-Arthritis Care & Research (Wiley)	11	1.5	1428	n.a.	n.a.	n.a.
	Arthritis Research & Therapy (BMC)	10	1.4	732	5.607	Q1	100
	Journal of Pain (Churchill Livingstone)	10	1.4	551	5.383	Q1	9.4
	Schmerz (Springer)	10	1.4	261	1.629	Q4	19.1

N° Doc. = number of documents; Cit. = number of citations; % Doc. = percentage of documents; JIF = journal impact factor; % O.A. = open access percentage; Q. = JIF quartile; n.a. = not applicable.

**Table 2:** Bradford’s Core and Zone 1 journals according to the number of citations.

Bradford’s Zone	Journals (Publishers)	N° Doc.	Cit.	%Cit.	JIF	Q.	%O.A.
Core	Journal of Rheumatology (The Journal of Rheumatology Publishing Co. Ltd.)	23	2469	9.4	5.346	Q2	1
	Pain (Lippincott Williams & Wilkins)	15	1557	5.9	7.926	Q1	16
	Arthritis & Rheumatism-arthritis Care & Research (Wiley)	11	1428	5.5	n.a.	n.a.	n.a.
	Annals of the Rheumatic Diseases (BMJ)	4	1323	5.1	27.973	Q1	38.7
	Jama (Journal of the American Medical Association)	2	1305	5	157.335	Q1	1.5
	Rheumatology International (Springer)	30	915	3.5	3.580	Q3	18.1
Zone I	Clinical & Experimental Rheumatology (Clinical & Experimental Rheumatology S.A.S.)	24	905	3.5	4.862	Q2	0
	Cochrane Database of Systematic Reviews (Wiley)	9	755	2.9	12.008	Q1	0.1
	Clinical Journal of Pain (Lippincott Williams & Wilkins)	12	741	2.8	3.423	Q2	9.3
	Arthritis Research & Therapy (BMC)	10	732	2.8	5.607	Q1	100
	Arthritis and Rheumatism (Wiley)	8	645	2.5	n.a.	n.a.	n.a.
	Journal of Pain (Churchill Livingstone)	10	551	2.1	5.383	Q1	9.4
	Arthritis Care & Research (Wiley)	15	548	2.1	5.178	Q2	13.5
	European Journal of Pain (Wiley)	8	542	2.1	3.651	Q2	27.3
	Best Practice & Research in Clinical Rheumatology (Elsevier)	4	518	2.	4.991	Q2	5.8
	Clinical Rheumatology (Springer)	15	445	1.7	3.650	Q3	10.8
	Scandinavian Journal of Rheumatology (Taylor & Francis)	6	430	1.6	3.057	Q3	23.8
	Archives of Physical Medicine and Rehabilitation (Elsevier)	12	395	1.5	4.060	Q1	6.2
	Current Pain and Headache Reports (Springer)	4	385	1.5	3.904	Q2	2.9
	Medicine and Science in Sports and Exercise (Lippincott Williams & Wilkins)	6	373	1.4	5.411	Q1	7
	Patient Education and Counseling (Elsevier)	2	351	1.3	3.467	Q1	13.5
	Rheumatology (Oxford Univ Press)	5	314	1.2	7.046	Q1	25.9

N° Doc. = number of documents; Cit. = number of citations; % Doc. = percentage of documents; JIF = journal impact factor; % O.A. = open access percentage; Q. = JIF quartile; n.a. = not applicable.

## Data Availability

Datasets are available through the corresponding author upon reasonable request.

## Appendix A

Most cited documents according to h-index (their Unique WOS ID).

WOS:000334306000022;	WOS:000392426900002;	WOS:000225070100028;
WOS:000254121100020;	WOS:000231194600002;	WOS:000259774100017;
WOS:000184239300011;	WOS:A1994NG01600026;	WOS:000181712100010;
WOS:000257602000015;	WOS:000086106000010;	WOS:000280227900024;
WOS:A1996UH09000004;	WOS:000263246400010;	WOS:000274481800003;
WOS:000250188700158;	WOS:000189245600007;	WOS:000223975100003;
WOS:000256503900032;	WOS:000089669300028;	WOS:000294470000007;
WOS:A1996UP53900019;	WOS:000286192000022;	WOS:000257602000007;
WOS:000233281800023;	WOS:000270691200032;	WOS:000079617700007;
WOS:000247116600033;	WOS:000281733300015;	WOS:000318907100033;
WOS:000226507700038;	WOS:000177243900015;	WOS:000182639000031;
WOS:000220764600006;	WOS:000188896400016;	WOS:000329188500042;
WOS:000250806200005;	WOS:000282826100038;	WOS:000171814800006;
WOS:A1992JF19400005;	WOS:000173317400004;	WOS:000283657300022;
WOS:000175430100030;	WOS:000289557100023;	WOS:000297712700007;
WOS:A1992HX73300011;	WOS:000236800400012;	WOS:A1992JA28300012;
WOS:000227270300008;	WOS:000224096900005;	WOS:000326483600017;
WOS:000372544500005;	WOS:000236800400013;	WOS:A1994NP65400003;
WOS:000300594200011;	WOS:000234216000006;	WOS:000075858700010;
WOS:000240985200002;	WOS:000322387300002;	WOS:A1996WM82400004;
WOS:000283185300004;	WOS:000320758700004;	WOS:000178625200011;
WOS:000252503400005;	WOS:000254206100004;	WOS:A1994PC52100010;
WOS:000347646000084;	WOS:000227976900022;	WOS:000177349900010;
WOS:000297350300001;	WOS:000184239300008;	WOS:000174214100028;
WOS:000184262700013;	WOS:000247823800003;	WOS:000222039400007;
WOS:000239339400007;	WOS:000263204300002;	WOS:000408840400040;
WOS:000255598800002;	WOS:000165597800033;	WOS:000174214100029;
